# Climatic thresholds associated with increased dengue incidence across climate zones in Peru (2001-2022)

**DOI:** 10.1016/j.joclim.2025.100546

**Published:** 2025-10-07

**Authors:** Wil Laura, Patricia Rivera, Cristina Davila, Pierre Velasquez, Susan Mateo, Carmen Yon, Betsabet Valderrama, Tania Ita Vargas

**Affiliations:** a Dirección de meteorología y evaluación ambiental atmosférica, Servicio Nacional de Meteorología e Hidrología. Lima, Perú; b Fenner school of environment and society, Australian National University. Canberra, Australia; c Universidade de São Paulo. São Paulo, Brasil; d Centro Nacional de Epidemiología, Prevención y Control de Enfermedades - CDC Perú. Lima, Perú; e Universidad Peruana Cayetano Heredia, Lima, Perú

**Keywords:** Climatic thresholds, Dengue incidence, Climate zones, Tree regression, Cross-correlation

## Abstract

**Introduction:**

Dengue fever has experienced a global rise in incidence and distribution, largely influenced by climate variability. Nonetheless, the specific climatic thresholds that trigger elevated dengue incidence rates, and the time lag between weather conditions and the case surges remain uncertain.

**Methods:**

Average weekly climate variables along with weekly dengue incidence rates from 2001 and 2022 were analyzed in districts grouped by its climate zone. A cross-correlation technique was used to determine the time lag between climatic variables and dengue incidence, while a fine-tuned regression tree model was utilized to identify climatic thresholds linked to the incidence of dengue surges.

**Results:**

Our findings indicate that specific combinations of climatic thresholds within each climate zone are associated with increased dengue incidence rate over a 20-week window, with air temperature having a frequent role. The North Coast had the highest average dengue incidence, with rates surging sevenfold when climatic thresholds were met (56 cases per 100,000 inhabitants). The Central Coast and North-High Rainforest zones experienced the most significant increases, with incidence rates rising 53-fold from baseline levels (0.13 cases and 2.4 cases per 100,000, respectively).

**Conclusions:**

This study identified distinct climatic thresholds that were met within a 20-week window preceding elevated dengue incidence rates in the six climate zones with the highest dengue prevalence in Peru. These insights enable dengue incidence rates forecasting weeks in advance using climatic data, offering a valuable tool for dengue mitigation and early intervention.

## 1 Introduction

Dengue is an acute arthropod-borne viral infection transmitted by mosquitoes of the genus Aedes [1], causing serious and increasing health impacts worldwide. Its transmission is closely related to climate variability [[2], [3], [4]]. The last decades have been successively warmer, with a global warming trend of 0.29°C observed over the past ten years [5]. In Peru, this trend is equally pronounced, as the average annual mean temperature anomalies over the past five years reached approximately 0.3°C with 2023 even surpassing 1°C above normal levels [6]. This warming trend has allowed more areas to be suitable for a higher reproduction of the dengue vector, leading to a higher incidence rate of the disease [[7], [8], [9]]. Over 100 nations have endemic dengue [2], and Peru is one of the nations that has faced reinfestation and increased disease spread in recent decades [10]. Notably, the number of dengue cases in Perú has grown exponentially in the last five years [11,12], rising from 15,287 cases in 2019 to 63,216 in 2022, with over a quarter of a million cases reported in 2023 [13,11,14], making it the second country in America with the most suspected cases of dengue [15]. This surge in cases underscores the substantial economic burden of dengue, whose average annual cost in Latin America is estimated at more than 3 billion (2015 US dollars) [16]. Peru has implemented dengue control measures, including enhanced epidemiological surveillance, improved healthcare services, and public awareness campaigns focused on fumigation, breeding sites elimination, and larval control [17]. While these efforts have helped mitigate the impact of the disease, the incidence rate continues to rise.

Progress has been made to confirm that an increase in dengue epidemics in Peru is related to variations in its climate conditions [18,19], driven by climate modes such as El Niño Southern Oscillation [20,21,18]. However, climate involves various spatial and temporal scales, interacting at different stages of dengue transmission. Therefore, the weekly range of climatic variables influencing dengue incidence rates in Peru remains undefined. The climate-dengue relationship is complex since climate influences mosquito dynamics, virus development, and human-mosquito interaction [22,23]. However, it is well-established that climatic conditions exponentially increase dengue transmission in Peru [18,24,25]. While temperature seasonality influences dengue in coastal and rainforest regions of Peru, the role of precipitation remains uncertain [18,19,21]. For instance, with an annual temperature of 26-29°C and relative humidity above 80%, a peak of dengue incidence was reached in districts of high risk of dengue in Peru [19]. Although annual temperature and humidity thresholds are known to influence dengue incidence rates, the specific weekly climatic thresholds and their time lags preceding epidemics in different Peruvian climatic zones, remain unknown. Understanding this insight would enable us to predict dengue incidence based on climate potentially aiding in the disease’s prevention weeks in advance.

To fill this gap, the present ecological study analyzes the climatic thresholds and time lags associated with elevated dengue incidence rates in high-prevalence climate zones. To identify the weekly lag when there is a strong association between the climate variable and the incidence rate, we used a cross-correlation technique [26]. And, to identify climatic thresholds, we employed a tree regression model to explain the weekly dengue incidence rate about weekly climatic variables [27]. Consequently, this study provides insights to anticipate high dengue epidemics based on weekly weather conditions.

## 2 Material and methods

### 2.1 Study area

**Fig. 1:**
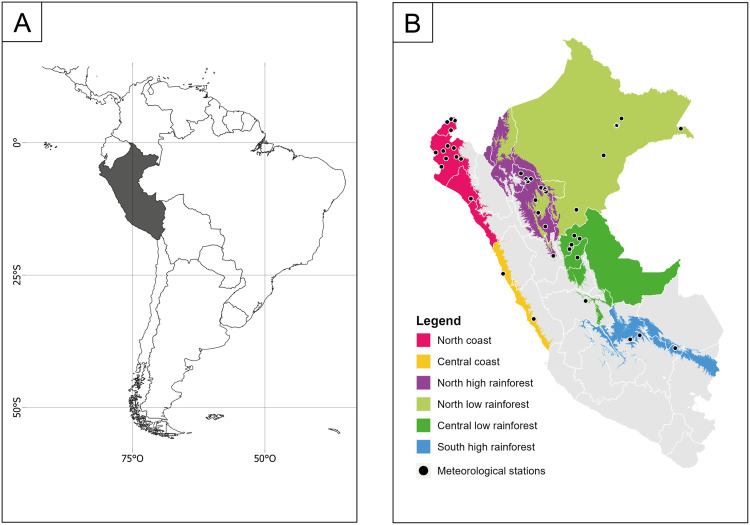

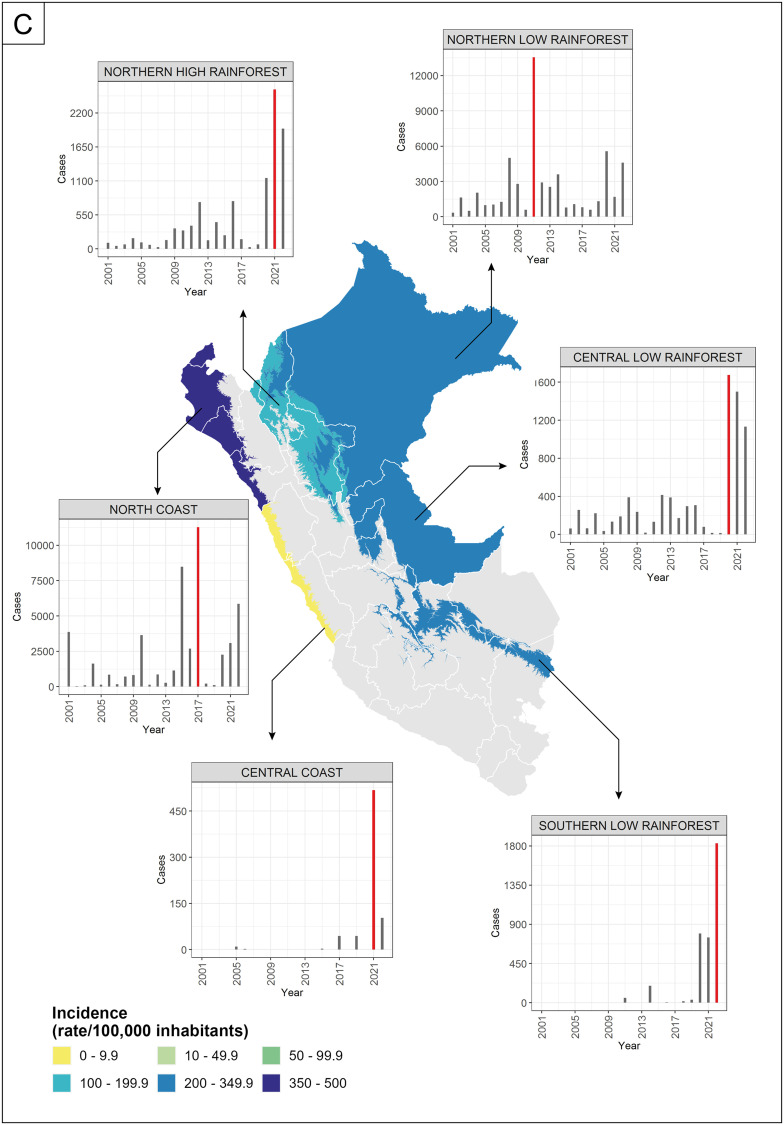
(a) Location of Peru in South America. (b) Location of meteorological stations in the six climate zones most affected by dengue in Peru. (c) Average annual accumulated incidence dengue rate by climate zone in colors (central map) and annual variability of dengue cases by climate zone (associated plots), where the red bar represents the year with the highest number of cases. The gray areas in Peru were not analyzed.

The study concentrated on six climatic zones in Peru where dengue has notably affected public health over the past two decades. Due to Peru’s high climatic diversity (38 climates) [28], the territory was classified into fifteen zones with similar climate conditions according to the latitudinal, longitudinal, altitudinal, and ecological characteristics [29]. The six climatic zones used were the North Coast, Central Coast, North-Low Rainforest, North-High Rainforest, Central-Low Rainforest, and South-High Rainforest, see Fig. 1.

Each climatic zone presents a particular set of climate conditions, see Table A1 in the supplementary files. The Northern Coast is characterized by an arid climate, with a year-round moisture deficit, a sparse seasonal precipitation pattern, and the warmest temperatures and heaviest rainfall on the Peruvian coast. Meanwhile, the Central Coast is the wettest part of the coast, with frequent cloud cover primarily in the spring and autumn of the southern hemisphere [29]. Warm and humid conditions characterized the North-Low Rainforest throughout the year, and the seasonal precipitation presents two peaks, in November and in March. On the other hand, the North-High Rainforest is located at higher altitudes, which by its geography presents higher amounts of precipitation up to 4,000 millimeters (mm) per year [29]. The Central-Low Rainforest experiences lower annual precipitation (3,000 mm) than the North-Low Rainforest, but it constantly maintains warm temperatures. Finally, the South-High Rainforest has Peru's highest precipitation levels, with annual totals of 5,000 mm [29]. Despite the varying climate conditions in each zone, dengue epidemics have occurred frequently across these areas.

### 2.2 Data

Dengue incidence rates were calculated in high-risk transmission districts of Peru, grouped by climatic zones. The study utilized a dataset of weekly dengue cases including probable and confirmed cases by district from 2001 to 2022 sourced from National Center for Epidemiology, Prevention and Disease Control of Peru (CDC-Peru) Health Ministry [30], available in the National Open Data Platform of the Peruvian Government. The weekly dengue incidence rate was defined as dengue cases per 100,000 inhabitants per week [31]. The population estimation by district was provided by the Peruvian National Institute of Statistics and Informatics. Only districts with at least 11 years of data were utilized.

The meteorological dataset, provided by the National Service of Meteorology and Hydrology of Peru (SENAMHI-Peru), was used to calculate the climate thresholds for its accurate representation of the climate conditions on the site with a weekly temporal resolution while accounting for its spatial representation limitations. It includes weekly average data on precipitation accumulation, relative humidity, maximum, minimum, and mean air temperature, as well as weekly averages of diurnal range, aligning with the period of dengue incidence rates.

41 meteorological stations spread across six climate zones were utilized (Table A2). Each station is in the district reporting dengue cases. The Central Coast had the fewest stations (two), with one station representing approximately 970 km^2^ limiting spatial climate representation variability. However, it provides greater precision compared to previous studies, such as Akter et al. [26], where one station covered ∼121,000 km^2^ of a single climatic zone. Station distribution included 13 in the North Coast, 2 in the Central Coast, 10 in the North-Low Rainforest, 7 in the North-High Rainforest, 6 in the Central-Low Rainforest, and 3 in the South-High Rainforest, see their locations in Fig. 1.

### 2.3 Methods

Determining the climatic thresholds associated with increased dengue incidence in the weeks before the disease involved a two-part process for each of the six climatic zones. All analyses were computed using R software version 4.3.1. The first part consisted of identifying the time lag in weeks where the climate variable and the incidence rate had their strongest association. For that purpose, a cross-correlation technique was used between the climatic variable and dengue incidence rate. The second part consisted of determining the climatic thresholds associated with a high dengue incidence rate weeks in advance. To achieve this, a regression tree model was employed, incorporating climatic variables and their time lags to predict dengue incidence rates.

Cross-correlation was employed for determining the optimal time lag in weeks at which the climate variable exhibits the strongest association with dengue incidence rates. This time lag corresponds to the maximum correlation, since the cross-correlation measures the similarity between the series of two variables as a function of their phases and amplitudes [32,33]. The cross-correlation between incidence rates and climate series was assessed over a range of lags from 0 to 20 weeks. Only correlations with statistical significance at a 95% confidence level were accepted. The climatic variables with their respective weekly lag and significant correlation were used to feed the regression tree model.

The regression tree model was developed to identify the climatic thresholds as predictors for estimating elevated dengue incidence rates. This model was selected due to its flexibility, robustness, and non-parametric nature, which make it well-suited for analyzing the complex interplay between dengue and climate data [26,27]. It splits the dataset into more homogeneous subgroups by choosing variables and threshold that results in the most significant reduction in variance based on nonlinear interactions between its covariates [34]. One of its most valuable features is its ability to identify the most significant climatic variables driving dengue incidence variability, as well as to determine the extent to which dengue incidence rates can rise under specific climatic thresholds.

The regression tree model was fine-tuned to improve estimates of the dengue incidence rate across the six climatic zones analyzed. This tuning process identified the optimal tree size with the lowest cross-validation error, enhancing predictive performance [35,36]. 48 regression tree models of varying sizes were compared by varying the model hyperparameter settings; see supplementary files for more details. Furthermore, the performance of the selected regression tree model in each climatic zone was evaluated by calculating the root mean square deviation, RMSE [37]. To achieve this, the dataset was randomly split into 70% for training the model and 30% for testing it. The 70/30 split strikes a good balance by allocating sufficient data for training while ensuring there is enough data to provide a reliable estimate of the model's performance.

## 3 Results

**Fig. 2:**
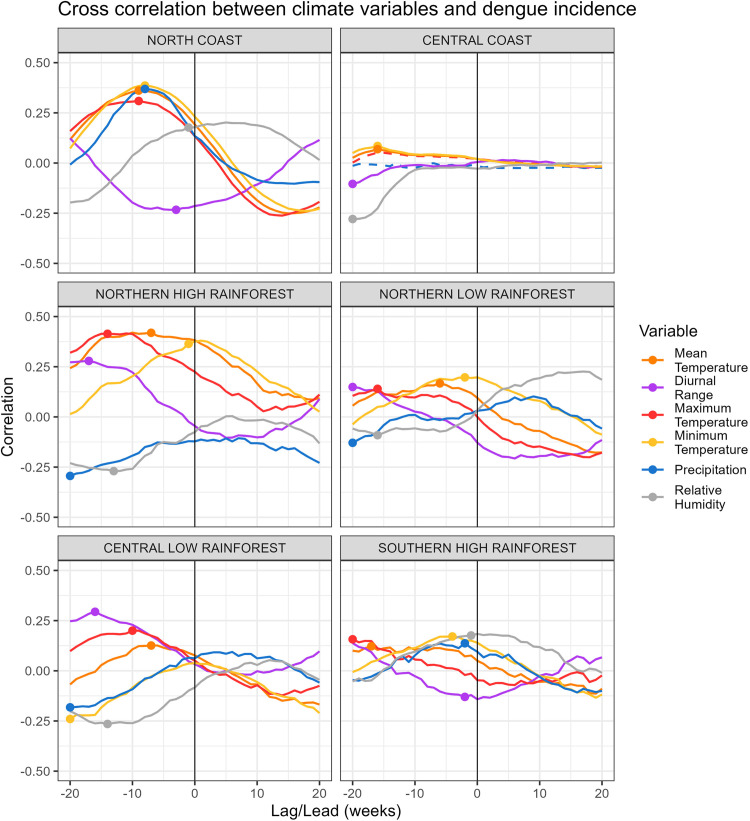
Weekly lag/lead cross-correlation between the dengue incidence rate and climatic variables: maximum temperature (red), minimum temperature (yellow), mean temperature (orange), diurnal range (violet), precipitation (blue), and relative humidity (gray) by climatic zone. Negative (positive) values on the x-axis represent the number of weeks that the climatic variables precede (follow) the peak incidence of dengue, with the dot marking the time lag exhibiting the strongest correlation. Significant (non-significant) correlations are shown in solid (dotted) lines.

**Fig. 3:**
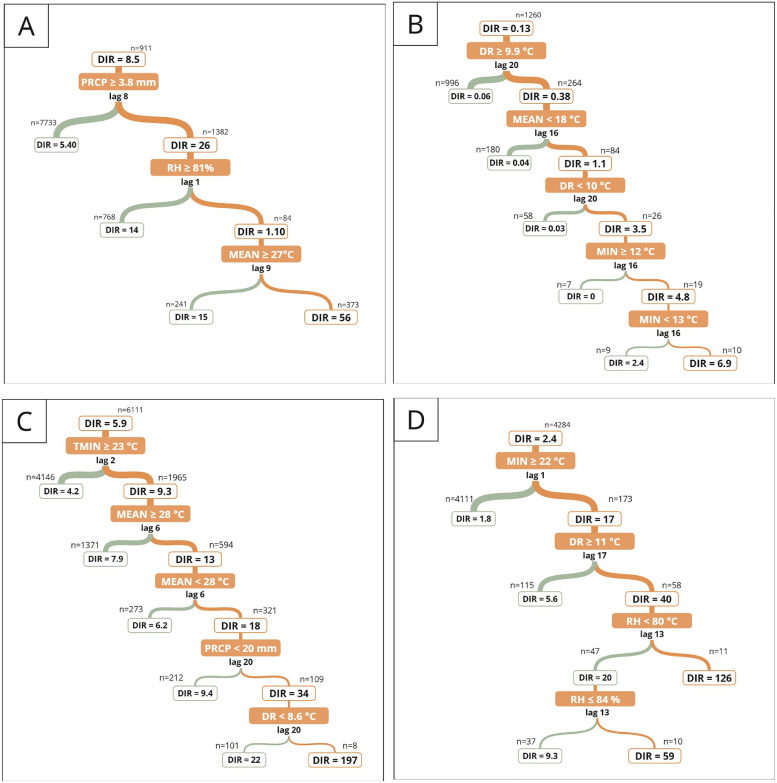

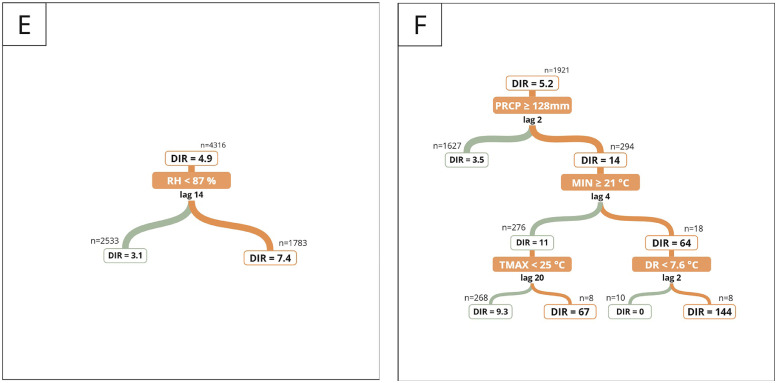
Climatic thresholds associated with dengue incidence rates across different climate zones. The subplots correspond to: (a) North Coast, (b) Central Coast, (c) North Low Rainforest, (d) North High Rainforest, (e) Central Low Rainforest, and (f) South High Rainforest. DIR stands for Dengue Incidence Rate, and n represents the number of weekly samples that met the threshold condition. Orange lines indicate conditions that were met, while green lines represent conditions that were not met.

Temperature, precipitation and relative humidity were associated with dengue incidence several weeks prior to the peak. Temperature variables showed a positive and statistically significant correlation with dengue incidence across all climatic zones, except for maximum air temperature on the central coast, where the correlation was not significant (Fig. 2). Meanwhile, the correlation with precipitation, relative humidity, and diurnal temperature range varied, being positive in some climatic zones and negative in others. The time lag with the highest association between dengue incidence and climatic variables varied for each climatic zone. See supplementary files for the time lag of each correlation. Each climate zone had a different tree regression size for explaining dengue incidence variability, with larger trees indicating more climatic thresholds interacting (Fig. 3). Furthermore, the leading predictor of dengue incidence rate varied by zone. In the North low and high rainforest zones, minimum temperature was the leading predictor, whereas in the North coast and South high rainforest zones, it was the precipitation. In the Central coast, the diurnal temperature range, while in the Central low rainforest, the relative humidity.

Dengue incidence rates surged 1.5 to 53 times above the general rate when specific climatic thresholds occurred weeks before an outbreak (Table 1). In zones where air temperature is the key factor, climatic conditions influence dengue incidence for up to 20 weeks, whereas in precipitation-dominated zones, incidence increases within 9 weeks. The description of all climatic thresholds that explain the variety of incidence rates by climatic zone is shown in supplementary files. The RMSE results (Table 1) supported the model’s reliability in predicting dengue variability based on the identified climatic thresholds.

**Table 1:** Model’s performance across climate zones. Characteristics observed when climatic thresholds associated with the highest incidence rates were reached. “Temp” refers to temperature. The incidence rate level classified the incidence based on its 33rd and 66th percentiles.

Climate zone	RMSE of model test	Climatic thresholds for highest incidence	Increase in dengue Incidence	Percentage of occurrence	Incidence rate level
North Coast	43.8	Precipitation ≥ 3.8 mm, 8 weeks before. Relative humidity ≥ 81%, 1 week before. Mean Temp ≥ 27°C, 9 weeks before.	7-fold its general rate (8.5 cases per 100,000)	4.1 %	High
Central Coast	2	Diurnal Temp Range ≥ 9.9°C, 20 weeks before. Mean Temp < 18°C, 16 weeks before. Minimum Temp ≥ 12°C & < 13°C, 16 weeks before.	53-fold its general rate (0.13 cases per 100,000)	0.8 %	High
North-Low Rainforest	17.2	Minimum Temp ≥ 23°C, 2 weeks before. Mean Temp = 28°C, 6 weeks before. Precipitation < 20 mm, 20 weeks before. Diurnal Temp Range < 8.6°C, 20 weeks before.	33-fold its general rate (5.9 cases per 100,000)	0.1 %	High
North-High Rainforest	10.1	Minimum Temp ≥ 22°C, 1 week before. Diurnal Temp Range ≥ 11°C, 17 weeks before. Relative humidity < 80%, 13 weeks before.	53-fold its general rate (2.4 cases per 100,000)	0.2 %	High
Central Low Rainforest	17.7	Relative humidity < 87%, 14 weeks before.	1.5-fold its general rate (4.9 cases per 100,000)	41 %	Moderate
South-High Rainforest	42.5	Precipitation ≥ 128 mm, 2 weeks before. Minimum Temp ≥ 21°C, 4 weeks before. Diurnal Temp Range < 7.6°C, 2 weeks before	28-fold its general rate (5.2 cases per 100,000)	0.4 %	High

Among the climatic zones, the north coast exhibited the highest dengue incidence rate and showed the strongest correlations between climatic variables and dengue incidence (Table 1 and Fig. 2). The incidence rate of dengue increased up to sixfold when the following conditions occurred: precipitation (≥3.8 mm) in the eighth week before the epidemic, high humidity (≥81%) during the week before the epidemic, and warm mean temperatures (≥27°C) in the ninth week (Table 1). This region experienced higher-than-average temperature and relative humidity thresholds, with anomalies of +1.7°C and +3.3% respectively (Table A1). Conversely, the precipitation threshold was below the average precipitation (6.1 mm). The central coast had a low incidence rate (Fig.s 1 and 3); however, this rate increased up to approximately 7 cases when conditions met the climatic thresholds outlined in Table 1. In this region, temperature was a critical factor, with the diurnal range surpassing normal levels by up to 1.5°C, whereas minimum and mean temperatures thresholds remained below average, with anomalies of –2.7°C and –1.9°C, respectively.

The most significant rise in dengue incidence due to weather conditions meeting the climatic thresholds was observed in the central coast and the north-high rainforest regions. In these areas, the rate increased 53-fold from their general incidence rate. However, the north-high rainforest zone had a higher rate overall, with 126 cases per 100,000 inhabitants. Precipitation was not a decisive factor for dengue incidence in this climatic zone, as rainfall remains consistently favorable year-round with minimal variation. In contrast, minimum temperature, diurnal temperature range, and relative humidity played a more significant role. On the other hand, the Central Low Rainforest, despite having a high annual dengue incidence rate (Fig. 1), showed the smallest increase in cases under the climatic thresholds, with a 1.5-fold increase and a weekly rate of 7 cases per 100,000 inhabitants (Table 1). In this zone, relative humidity was the sole significant climate variable explaining the variability in dengue incidence, although this threshold could only differentiate between low and moderate incidence rates. The relative humidity threshold was 1.1% above normal levels. These findings suggest that another major factor is affecting dengue incidence in this area. Similarities were found in the values of temperature thresholds between the zones north and south of the rainforest. Weeks with a minimum temperature of at least 21°C, which correspond to positive anomalies of +1.2°C in North Low Rainforest, +2.8°C in North High Rainforest, and +2.5°C in South High Rainforest, were associated with high dengue incidence rates (Table 1 and A1).

## 4 Discussion

Climate thresholds influencing dengue incidence varied across the analyzed zones. This study identified temperature, precipitation, and relative humidity thresholds, with a time lag of up to 20 weeks, associated with high dengue incidence, varying across climate zones. When the specific combination of climatic thresholds unique to each zone was met, dengue incidence rates increased, with central coast and north-high rainforest regions experiencing up to 53 times the average incidence. Air temperature emerged as the most consistent predictor, associated with high dengue incidence in five of six climate zones examined. However, the interplay of meteorological conditions over a short period (20 weeks) played a critical role in driving high dengue incidence rates.

Previous research has demonstrated the role of climatic conditions in shaping dengue incidence seasonality [38,39]. Building upon this evidence, our work identified temperature related thresholds, specifically mean, minimum, and diurnal temperature range, as primary drivers of dengue incidence variability in Peru. Temperature directly impacts on both vector and viral dynamics, influencing factors such as egg hatching rates, larval development time, adult vector survival, and the extrinsic incubation period [[40], [41], [42], [43], [44], [45], [46]]. However, temperature was not the only key variable for predicting dengue incidence; precipitation also played a crucial role, notably, in Peru's arid north coast region, where low precipitation thresholds triggered outbreaks. As standing water serves as breeding sites for mosquitoes [47], even small-scale breeding sites (e.g., plastic drums) can substantially amplify vector populations [40]. Beyond climatic conditions, socio-economic conditions, human behavior, and educational levels vary across the regions and contribute to dengue risk [45,46,48]. These factors often become more pronounced during strong El Niño events. El Niño typically causes significant rainfall in northern Peru, above-average temperatures across much of South America and flooding along the tropical west coast and south-eastern South America [[49], [50], [51]]. For instance, during the 2017 coastal El Niño event, Peru recorded its highest annual number of dengue cases [52].

Comparatively, our findings align with previous studies that have established a strong relationship between climate variability and dengue transmission worldwide [53]. Research in Australia, Brazil and Taiwan identified climatic thresholds triggering dengue incidence up to five months in advance [26,27,[54], [55], [56]], consistent with our 4.5 month (20 week) predictive window. Notably, precipitation and temperature emerged as key drivers. These parallels underscore the global relevance of climate-based dengue prediction models, despite regional variations in transmission patterns. For instance, the tropical climate of Queensland, Australia, and the arid climate of Peru's north coast share a mean temperature threshold of ≥27°C. However, the lead times differ: 2 months in Queensland [26] and 9 weeks in northern Peru. Additionally, a global study of 94 islands determined that increases in dengue transmission required a minimum monthly temperature exceeding 14.8°C (95% CI: 12.4–16.6°C) [57]. Our results identified a similar minimum temperature threshold of 12°C for the central coast of Peru, though the timing and intensity of dengue incidence varied across regions.

This study acknowledges certain limitations. In terms of the climate data, the selection of weather stations to represent climate zones should be carefully considered. While the climatic zone serves as the spatial unit of analysis, climatic thresholds may vary at higher spatial resolutions due to local climate conditions. Regarding the methodology, confounding factors affecting dengue incidence rates, such as human interventions, including dengue control measures, were not accounted for. Addressing these factors could improve the reliability of climate influence on dengue incidence. To better capture the complexity of dengue incidence, epidemiological models that incorporate both climatic and non-climatic factors should be developed.

Our results identify key climatic variables, critical thresholds, and lead times that provide actionable inputs for early warning systems in Peru, enabling proactive responses to dengue outbreaks. This study highlights the importance of considering all climate threshold combinations that contribute to increased dengue incidence, including those occurring more frequently, even when predicting moderate outbreak levels. Furthermore, these insights raise an important question about which regions in Peru may become more susceptible to dengue outbreaks in the following decades as climate change progresses. Understanding the climatic thresholds that precede spikes in dengue incidence can significantly prevent outbreaks in the most affected climatic zones. These thresholds can be integrated into health and weather monitoring services, improving early detection and response.

## 5 Conclusion

This study underscores the critical role of climatic thresholds in influencing dengue incidence across diverse climate zones in Peru. Our findings revealed significant interactions among weekly averages of temperature, precipitation, and relative humidity within a 4.5-month period preceding elevated dengue incidence rates. The climatic variables influencing dengue incidence and the number of thresholds defining optimal weekly weather conditions varied across climatic zones. Despite the diversity of results, air temperature was the most frequent predictor across climate zones, explaining a high dengue incidence rate. Among the six climatic zones analyzed, the north coast exhibited the highest average weekly dengue incidence, with rates increasing seven-fold when climatic threshold conditions were met. The central coast and north-high rainforest zones experienced the greatest increase in dengue incidence, with rates rising 53-fold when the climatic thresholds were met. Despite the complex interaction of factors driving dengue outbreaks, this research provides a route for estimating dengue incidence weeks in advance using climatic thresholds. This approach aims to reduce the impact of dengue in the most affected climatic zones of Peru.

## CRediT authorship contribution statement

**Wil Laura:** Writing – review & editing, Writing – original draft, Visualization, Validation, Software, Methodology, Investigation, Formal analysis, Conceptualization. **Patricia Rivera:** Writing – review & editing, Visualization, Supervision, Formal analysis. **Cristina Davila:** Visualization, Formal analysis, Conceptualization. **Pierre Velasquez:** Writing – review & editing, Methodology. **Susan Mateo:** Writing – review & editing, Methodology. **Carmen Yon:** Writing – review & editing. **Betsabet Valderrama:** Writing – review & editing. **Tania Ita Vargas:** Writing – review & editing.

## Declaration of competing interest

The authors declare that they have no known competing financial interests or personal relationships that could have appeared to influence the work reported in this paper.

## Appendix Supplementary materials

Image, application 1
