# Supplementary material for: Climatic thresholds associated with increased dengue incidence across climate zones in Peru (2001-2022)
Source: J Clim Chang Health. 2025 Oct 7;26:100546. doi: 10.1016/j.joclim.2025.100546 (PMC12851297; doi:10.1016/j.joclim.2025.100546)
Supplement: Supplementary file 1 [file mmc1.docx]

# **Appendix – Supplementary material**

Table A1. Climate normals considering 3 decades of average from 1991 to 2020, by climate zone

| **Sector** | **Precipitation (mm)** | **Maximum temp (°C)** | **Minimum**  **Temp (°C)** | **Mean**  **Temp (°C)** | **Relative humidity (%)** * | **Diurnal temp range (°C)** |
| --- | --- | --- | --- | --- | --- | --- |
| North Coast | 6.1 | 30.7 | 19.8 | 25.3 | 77.7 | 10.9 |
| Central Coast | 0.1 | 24.1 | 15.7 | 19.9 | 86.1 | 8.4 |
| North-High Rainforest | 35.7 | 29.1 | 19.2 | 24.1 | 84.4 | 9.9 |
| North-Low Rainforest | 34.6 | 32.2 | 21.8 | 27 | 85.2 | 10.5 |
| Central-Low Rainforest | 46.8 | 31.6 | 20.7 | 26.2 | 85.9 | 11 |
| South-High Rainforest | 47.5 | 29.7 | 18.5 | 24.1 | 85.2 | 11.2 |

(*) Normals calculated considering the study period (2001-2020) due to the lack of historical information for the variable

Table A2. SENAMHI meteorological stations used in the study for each climate zone

| **Climate zone** | **Station's name** | **Departments** | **Latitude** | **Longitude** |
| --- | --- | --- | --- | --- |
| North Coast | Cabo Inga | Tumbes | -4.0 | -80.4 |
|  | Cayalti | Lambayeque | -6.9 | -79.5 |
|  | Chulucanas | Piura | -5.1 | -80.2 |
|  | Chusis | Piura | -5.5 | -80.8 |
|  | La Cruz | Tumbes | -3.6 | -80.6 |
|  | La Esperanza | Piura | -4.9 | -81.1 |
|  | Lancones | Piura | -4.6 | -80.5 |
|  | Mallares | Piura | -4.9 | -80.7 |
|  | Miraflores | Piura | -5.2 | -80.6 |
|  | Morropon | Piura | -5.2 | -80.0 |
|  | Papayal | Tumbes | -3.6 | -80.2 |
|  | Partidor | Piura | -4.7 | -80.3 |
|  | Puerto Pizarro | Tumbes | -3.5 | -80.4 |
| Central Coast | Huarmey | Ancash | -10.1 | -78.2 |
|  | Ñaña | Lima | -12.0 | -76.8 |
| North-High Rainforest | Lamas | San Martin | -6.4 | -76.5 |
|  | Moyobamba | San Martin | -6.0 | -77.0 |
|  | Naranjillo | San Martin | -5.8 | -77.4 |
|  | Rioja | San Martin | -6.0 | -77.2 |
|  | Soritor | San Martin | -6.1 | -77.1 |
|  | Tananta | San Martin | -8.1 | -76.3 |
|  | Tingo Maria | Huanuco | -9.3 | -76.0 |
| North-Low Rainforest | Caballococha | Loreto | -3.9 | -70.5 |
|  | Puerto Almendra | Loreto | -3.8 | -73.3 |
|  | Amazonas | Loreto | -3.8 | -73.3 |
|  | Bellavista | San Martin | -3.5 | -73.1 |
|  | Campanilla | San Martin | -7.5 | -76.6 |
|  | Contamana | Loreto | -7.4 | -75.0 |
|  | El Porvenir | San Martin | -6.6 | -76.3 |
|  | Requena | Loreto | -5.0 | -73.8 |
|  | Saposoa | San Martin | -6.9 | -76.8 |
|  | Tarapoto | San Martin | -6.5 | -76.4 |
| Central-Low Rainforest | Aguaytia | Ucayali | -9.0 | -75.3 |
|  | El Maronal | Ucayali | -8.4 | -75.1 |
|  | Las Palmeras De Ucayali | Ucayali | -8.6 | -74.9 |
|  | Puerto Inca | Huánuco | -9.4 | -75.0 |
|  | San Alejandro | Ucayali | -8.8 | -75.2 |
|  | Satipo | Junin | -11.2 | -74.6 |
| South-High Rainforest | Quebrada Yanatile | Cusco | -12.7 | -72.3 |
|  | Quillabamba | Cusco | -12.9 | -72.7 |
|  | Quincemil | Cusco | -13.2 | -70.8 |

**Spectrum of hyperparameter values**

The hyperparameters of the regression tree model modified and tested to get the optimum tree size were the minimum percentage by leaf and the maximum depth of the tree. A total of 48 tree models were tested. The minimum percentage by leave varied from 5% to 20% and the maximum depth of the tree varied from 3 to 5 levels. The error evaluation was done in a 10-fold cross-validation. For instance, the variation of errors according to different tree sizes for central coast zone can be seen in the following figure. All the analyses were performed using R software (version 4.4.0) and the part package was used for regression tree analysis.


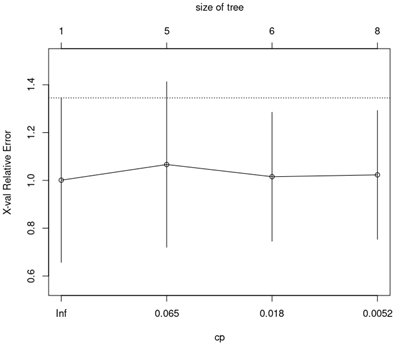


Figure A1. Cross-validation error for different sizes of tree model to the central coast.

Table A3. Metrics of the tuned regression tree model for each climatic zone. Minsplit means the minimum number of data points required to attempt a split before it is forced to create a terminal node. Maxdepth means the maximum number of internal nodes between the root node and the terminal nodes.

| Minsplit | Maxdepth | error | Climatic zone | RMSE Tree model |
| --- | --- | --- | --- | --- |
| 8 | 3 | 0.96 | North Coast | 43.8 |
| 19 | 5 | 0.84 | Central Coast | 2 |
| 14 | 5 | 1.0 | North-Low Rainforest | 17.2 |
| 17 | 4 | 0.98 | North-High Rainforest | 10.1 |
| 7 | 5 | 0.98 | Central-Low Rainforest | 17.7 |
| 11 | 3 | 0.96 | South-High Rainforest | 42.5 |

Table A.4. Time lag for each correlation of climate variables with dengue incidence for climatic zones. NS means that the correlation was not significant.

| **Climatic Zone** | **Maximum temp** | **Minimum**  **temp** | **Mean**  **temp** | **Precipitation** | **Relative humidity** | **Diurnal temp range** |
| --- | --- | --- | --- | --- | --- | --- |
| North Coast | 9 | 8 | 9 | 8 | 1 | 3 |
| Central Coast | NS | 16 | 16 | NS | 20 | 20 |
| North-Low Rainforest | 16 | 2 | 6 | 20 | 16 | 20 |
| North-High Rainforest | 14 | 1 | 7 | 20 | 13 | 17 |
| Central Low Rainforest | 10 | 20 | 7 | 20 | 14 | 16 |
| South-High Rainforest | 20 | 4 | 17 | 2 | 1 | 2 |
| Average | 14 | 9 | 10 | 14 | 11 | 13 |

**Climatic thresholds that explain the dengue incidence variability by each climate zone**

North Coast

| **Dengue incidence (cases x 100,000 inhabitants)** | **Climatic thresholds** | **Classification of incidence** |
| --- | --- | --- |
| 5.4 | prcp_lag_8 < 3.8 | Low |
| 14 | prcp_lag_8 >= 3.8 & hrel_lag_1 < 81 | Moderate |
| 15.1 | prcp_lag_8 >= 3.8 & hrel_lag_1 >= 81 & tmean_lag_9 < 27 | Moderate |
| 56.4 | prcp_lag_8 >= 3.8 & hrel_lag_1 >= 81 & tmean_lag_9 >= 27 | High |

Central Coast

| **Dengue incidence (cases x 100,000 inhabitants)** | **Climatic thresholds** | **Classification of incidence** |
| --- | --- | --- |
| 0 | rdiu_lag_20 is 9.9 to 10.3 & tmean_lag_16 < 18 & tmin_lag_16 < 12 | No cases |
| 0.031 | rdiu_lag_20 >= 10.3 & tmean_lag_16 < 18 | Low |
| 0.04 | rdiu_lag_20 >= 9.9 & tmean_lag_16 >= 18 | Low |
| 0.064 | rdiu_lag_20 < 9.9 | Low |
| 2.4 | rdiu_lag_20 is 9.9 to 10.3 & tmean_lag_16 < 18 & tmin_lag_16 >= 13 | Moderate |
| 6.9 | rdiu_lag_20 is 9.9 to 10.3 & tmean_lag_16 < 18 & tmin_lag_16 is 12 to 13 | High |

North-Low Rainforest

| **Dengue incidence (cases x 100,000 inhabitants)** | **Climatic thresholds** | **Classification of incidence** |
| --- | --- | --- |
| 4.2 | tmin_lag_2 < 23 | Moderate |
| 6.2 | tmin_lag_2 >= 23 & tmean_lag_6 >= 28 | Moderate |
| 7.9 | tmin_lag_2 >= 23 & tmean_lag_6 < 28 | Moderate |
| 9.4 | tmin_lag_2 >= 23 & tmean_lag_6 is 28 to 28 & prcp_lag_20 >= 20 | Moderate |
| 21.5 | tmin_lag_2 >= 23 & tmean_lag_6 is 28 to 28 & prcp_lag_20 < 20 & rdiu_lag_20 >= 8.6 | High |
| 197 | tmin_lag_2 >= 23 & tmean_lag_6 is 28 to 28 & prcp_lag_20 < 20 & rdiu_lag_20 < 8.6 | High |

North-High Rainforest

| **Dengue incidence (cases x 100,000 inhabitants)** | **Climatic thresholds** | **Classification of incidence** |
| --- | --- | --- |
| 1.8 | tmin_lag_1 < 22 | Low |
| 5.6 | tmin_lag_1 >= 22 & rdiu_lag_17 < 11 | Moderate |
| 9.3 | tmin_lag_1 >= 22 & rdiu_lag_17 >= 11 & hrel_lag_13 is 80 to 84 | Ligh |
| 59 | tmin_lag_1 >= 22 & rdiu_lag_17 >= 11 & hrel_lag_13 >= 84 | High |
| 125.5 | tmin_lag_1 >= 22 & rdiu_lag_17 >= 11 & hrel_lag_13 < 80 | High |

Central-Low Rainforest

| **Dengue incidence (cases x 100,000 inhabitants)** | **Climatic thresholds** | **Classification of incidence** |
| --- | --- | --- |
| 3.1 | hrel_lag_14 >= 87 | Low |
| 7.4 | hrel_lag_14 < 87 | Moderate |

South-High Rainforest

| **Dengue incidence (cases x 100,000 inhabitants)** | **Climatic thresholds** | **Classification of incidence** |
| --- | --- | --- |
| 0 | prcp_lag_2 >= 128 & tmin_lag_4 >= 21 & rdiu_lag_2 >= 7.6 | Low |
| 3.5 | prcp_lag_2 < 128 | Low |
| 9.3 | prcp_lag_2 >= 128 & tmin_lag_4 < 21 & tmax_lag_20 >= 25 | Low |
| 67.2 | prcp_lag_2 >= 128 & tmin_lag_4 < 21 & tmax_lag_20 < 25 | High |
| 144.5 | prcp_lag_2 >= 128 & tmin_lag_4 >= 21 & rdiu_lag_2 < 7.6 | High |
